# Correction: The role of brain health and resilience in reshaping trajectories of late-life neuropsychiatric disorders

**DOI:** 10.1038/s41386-026-02384-4

**Published:** 2026-03-13

**Authors:** Helen Lavretsky, Sahib Khalsa, Hanadi Ajam Oughli, Agustin Ibanez, Josefina Cruzat, Emmeline Edwards, Paul Newhouse, Claudio L. A. Bassetti, Indrit Begue, Andrea S. Winkler, Dilip V. Jeste, Harris A. Eyre

**Affiliations:** 1 Department of Psychiatry and Biobehavioral Sciences, Semel Institute for Neuroscience and Human Behavior, David Geffen School of Medicine, University of California at Los Angeles, Los Angeles, CA USA; 2 Latin American Brain Health Institute (BrainLat), Universidad Adolfo Ibañez, Santiago, Chile; 3 Global Brain Health Institute (GBHI), Trinity College Dublin, Dublin, Ireland; 4 World Women in Neuroscience, 501(c)(3); retired as of 5/31/2025, National Center for Complementary and Integrative Health (until May 27, 2025), Ellicott city, MD USA; 5 Department of Psychiatry and Behavioral Sciences, Center for Cognitive Medicine, Vanderbilt University Medical Center, Nashville, TN USA; 6 Department of Neurology, Medical Faculty, University of Bern, Inselspital, Bern, Switzerland; 7 Department of Psychiatry, Laboratory for Neuroimaging and Translational Psychiatry, University of Geneva and University Hospitals of Geneva, Geneva, Switzerland; 8 Department of Neurology, TUM University Hospital, and Center for Global Health, TUM School of Medicine and Health, Technical University of Munich (TUM), Munich, Germany; 9 Department of Global Health and Community Medicine, Institute of Health and Society, and Founding Director of the Center for Global Health, University of Oslo, Oslo, Norway; 10 Social Determinants of Health Network, La Jolla, CA USA; 11 Department of Psychological Sciences, Office of Innovation, Center for Health, Baker Institute for Public Policy, School of Engineering, Rice University, Houston, TX USA

**Keywords:** Medical research, Neuroscience

Correction to: *Neuropsychopharmacology* 10.1038/s41386-026-02332-2, published online 28 January 2026

The incorrect version of Figure 1 was published where midfulness is listed instead of mindfulness and Senescence was misspelled.

Figure 1 should appear as:
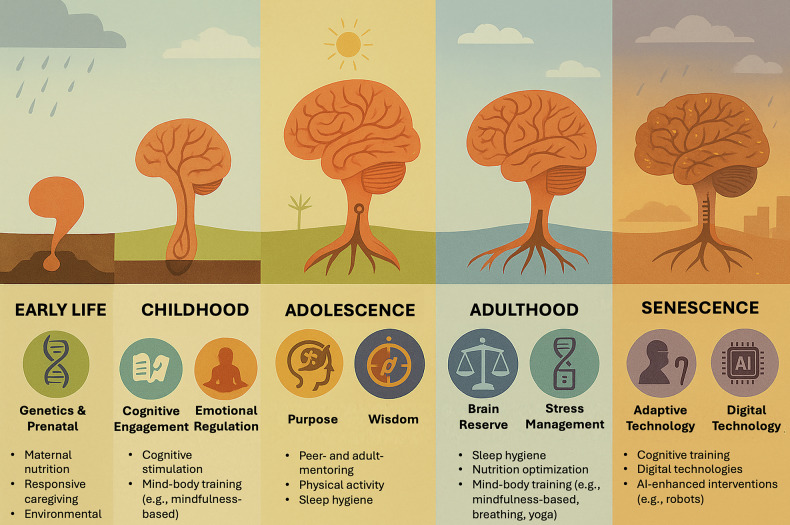


The original article has been corrected.

